# Phosphorylation of Threonine 175 Tau in the Induction of Tau Pathology in Amyotrophic Lateral Sclerosis—Frontotemporal Spectrum Disorder (ALS-FTSD). A Review

**DOI:** 10.3389/fnins.2018.00259

**Published:** 2018-04-20

**Authors:** Alexander J. Moszczynski, Matthew A. Hintermayer, Michael J. Strong

**Affiliations:** ^1^Molecular Medicine Research Group, Schulich School of Medicine & Dentistry, Robarts Research Institute, Western University, London, ON, Canada; ^2^Department of Clinical Neurological Sciences, Schulich School of Medicine & Dentistry, Western University, London, ON, Canada

**Keywords:** amyotrophic lateral sclerosis, chronic traumatic encephalopathy, frontotemporal dementia, TDP-43, microtubule associated tau protein

## Abstract

Approximately 50–60% of all patients with amyotrophic lateral sclerosis (ALS) will develop a deficit of frontotemporal function, ranging from frontotemporal dementia (FTD) to one or more deficits of neuropsychological, speech or language function which are collectively known as the frontotemporal spectrum disorders of ALS (ALS-FTSD). While the neuropathology underlying these disorders is most consistent with a widespread alteration in the metabolism of transactive response DNA-binding protein 43 (TDP-43), in both ALS with cognitive impairment (ALSci) and ALS with FTD (ALS-FTD; also known as MND-FTD) there is evidence for alterations in the metabolism of the microtubule associated protein tau. This alteration in tau metabolism is characterized by pathological phosphorylation at residue Thr^175^ (pThr^175^ tau) which *in vitro* is associated with activation of GSK3β (pTyr^216^GSK3β), phosphorylation of Thr^231^tau, and the formation of cytoplasmic inclusions with increased rates of cell death. This putative pathway of pThr^175^ induction of pThr^231^ and the formation of pathogenic tau inclusions has been recently shown to span a broad range of tauopathies, including chronic traumatic encephalopathy (CTE) and CTE in association with ALS (CTE-ALS). This pathway can be experimentally triggered through a moderate traumatic brain injury, suggesting that it is a primary neuropathological event and not secondary to a more widespread neuronal dysfunction. In this review, we discuss the neuropathological underpinnings of the postulate that ALS is associated with a tauopathy which manifests as a FTSD, and examine possible mechanisms by which phosphorylation at Thr^175^tau is induced. We hypothesize that this might lead to an unfolding of the hairpin structure of tau, activation of GSK3β and pathological tau fibril formation through the induction of *cis*-Thr^231^ tau conformers. A potential role of TDP-43 acting synergistically with pathological tau metabolism is proposed.

## Introduction

Amyotrophic lateral sclerosis (ALS, Lou Gehrig's Disease) has classically been considered to be a disorder purely of the descending supraspinal and lower motor neurons, the net result of which is an insidiously progressive degenerative process culminating in death within 3–5 years following symptom onset for the majority of patients (Charcot and Joffroy, 1869; Strong, 2003). However, the concept that ALS is a pure motor disorder has been gradually replaced by one in which it is recognized that between 50 and 60% of all ALS patients will have a concurrent disorder of frontotemporal function (Strong, 2008; Elamin et al., 2011; Phukan et al., 2012; Oh et al., 2014; Montuschi et al., 2015; Strong et al., 2017). Although the literature historically contained a number of individual ALS case reports in whom a concurrent neuropsychological or behavioral disorder had been identified, the recognition that a significant proportion of ALS patients could also have a frank dementia is more recent (Hudson, 1981; Bak and Hodges, 2001). It is now recognized that ALS can include a broad range of neuropsychological, speech, language, or behavioral pathologies that have recently been termed “amyotrophic lateral sclerosis – frontotemporal spectrum disorder (ALS-FTSD)” (Strong et al., 2017). ALS-FTSD includes impairments in cognition (ALSci), behavioral dysfunction (ALSbi), a dysexecutive syndrome (ALScbi), or a frontotemporal dementia (ALS-FTD) meeting the Neary or Hodges criteria (Neary et al., 1998; Hodges and Miller, 2001; Rascovsky et al., 2007).

As our understanding of the clinical phenomenology of ALS-FTSD has evolved, so too has our understanding of its pathobiology. In contemporary nomenclature, the frontotemporal lobar degenerations (FTLD) are categorized into three neuropathological subtypes depending on the pattern of pathological protein deposition that is observed: FTLD-tau for those in which a tauopathy [pathological inclusions consisting of the microtubule associated protein tau (tau)] is clearly evident; FTLD-TDP for those in which transactive response DNA-binding protein of 43 kDa (TDP-43) deposition as neuronal or glial inclusions is the hallmark; and, FTLD-FUS for a small proportion of cases in which the DNA/RNA binding protein, fused in sarcoma (FUS), is deposited. For a minority of cases, none of these markers are evident, but there is evidence of widespread protein ubiquitin conjugation, suggesting a disorder of the ubiquitin-proteasome system (UPS). In this latter subgroup, the terminology FTLD-UPS is applied. Finally, an exceptionally rare subgroup will demonstrate a FTLD in which the molecular pathology (i.e., tau, TDP-43, FUS or ubiquitin) is not known, in which case the terminology of FTLD-NOS (not otherwise specified) is applied (Mackenzie et al., 2011; Irwin et al., 2015).

In its most florid form, ALS-FTSD bears all of the neuropathological features of a FTLD in which there is a strong positive correlation between the degree of cognitive dysfunction and the extent of pathological intraneuronal deposition of TDP-43 (Cykowski et al., 2017). Typically observed as a predominantly nuclear protein, in ALS and under conditions of neuronal stress or injury, TDP-43 undergoes a marked upregulation in its expression and adopts a predominantly cytosolic localization (Arai et al., 2006; Neumann et al., 2006; Mackenzie et al., 2011). This applies to both motor neurons and cortical neurons where the latter forms the basis for characterizing the FTLD of ALS as FTLD-TDP. This has been taken therefore to imply that the frontotemporal dysfunction of ALS cannot be grounded in alterations in the metabolism of tau, or potentially as an overlap syndrome. In this review, we will examine the evidence for alterations in tau metabolism in ALSci, and provide the basis for the consideration of this disorder as a tauopathy that is tightly integrated with the dysmetabolism of TDP-43.

## ALS-FTSD: clinical & neuroimaging characterization

The presence of frontotemporal dysfunction in an ALS patient is a negative prognostic indicator, heralding a significant reduction in survivorship. This is particularly true for those individuals in which the primary manifestation is either a dysexecutive syndrome or behavioral impairment (Olney et al., 2005; Elamin et al., 2011; Hu et al., 2013). While current epidemiological studies consider a range of prognostic factors that can have an impact on survivorship such as age of onset, rate of deterioration as measured by the ALSFRS–R Total Score, diagnostic delay, age at diagnosis, and metabolic markers, it is noteworthy that few account for the presence or absence of neuropsychological, speech or language dysfunction as a significant prognostic variable (Chiò et al., 2009; Lunetta et al., 2015; Couratier et al., 2016). In part, this may represent a bias introduced because of the complexity of detecting neuropsychological, speech, language, or behavioral impairments (Farhan et al., 2017). Because of this, the Strong criteria (2009) have been recently revised to increase the simplicity and applicability of these international consensus criteria (Strong et al., 2009, 2017).

Revision of the Strong criteria was also driven by the need to broaden the definition of frontotemporal spectrum disorder in ALS to include deficits in social cognition, including deficits in Theory of Mind (ToM). ToM refers specifically to the capacity to attribute independent mental states to others, and can be divided into an affective and cognitive component. The affective component of ToM refers to one's ability to make inferences regarding the emotions and feelings of others, whereas the cognitive component refers to the ability to infer others' intentions and beliefs (Shamay-Tsoory et al., 2009; Adenzato et al., 2010; Poletti et al., 2012). Deficits in both social cognition and ToM, localizing to the mesial frontal/anterior cingulate, are present in a significant proportion of ALS patients, even when there is no clear evidence to suggest more widespread higher-order cortical dysfunction (Meier et al., 2010; Girardi et al., 2011; Poletti et al., 2012; Cerami et al., 2014; van der Hulst et al., 2015). This suggests that deficits in social cognition and ToM might be the earliest harbinger of frontotemporal dysfunction in ALS.

The concept of non-motor dysfunction in ALS being reflective of a widespread higher cortical impairment—with a degree of preponderance to the frontal lobes, particularly the mesial frontal cortex—is supported by a broad array of neuroimaging studies (Abrahams et al., 2004; Lillo et al., 2010; Agosta et al., 2011, 2013; Goldstein et al., 2011; Prudlo et al., 2012; Ambikairajah et al., 2014). Such studies need to be interpreted in the light of underlying genetic traits of the individuals being studied, as it is increasingly clear that ALS is also syndromic with the clinical and molecular phenotype being driven by an ever-increasing array of known genetic variants (Strong, 2001, 2017; Turner and Verstraete, 2015). The contributions of neuroimaging have extended beyond identifying the general degree of cerebral atrophy accompanying frontotemporal syndromes, as visualized with either computed tomography (CT) or magnetic resonance imaging (MRI). Neuroimaging platforms such as single positron emission computerized tomography (SPECT) (Neary et al., 1990), MRI-based measures of functional connectivity (Douaud et al., 2011), and advanced structural MRI sequences that define subcortical frontotemporal white matter tract projection pathology (Agosta et al., 2016) can now provide an evaluation of the extent of loss of functional integrity. Positron emission tomography (PET) has proven invaluable in our understanding of the anatomic and cellular extent of the pathobiology of ALS-FTSD, including the involvement of non-neuronal cells in the disease process (Cistaro et al., 2012, 2014). Increasingly, these neuroimaging modalities are being linked to understanding the molecular pathology of ALS, such as the degree of deeper cortical structures and cerebellar pathology evident in those ALS patients carrying pathological hexanucleotide expansions in *C9orf72*, even in the presymptomatic stages (Mahoney et al., 2012; Bede et al., 2013; Rohrer et al., 2015; Walhout et al., 2015).

Our interest in the pathology of the anterior cingulate gyrus in ALS, and ultimately the importance of alterations in metabolism of the microtubule-associated protein tau, was driven by a broad array of evidence suggesting that alterations in verbal praxis and fluency were key harbingers of higher cortical dysfunction in the disease (Strong et al., 1996). In a prospective study designed to assess the degree of neuronal loss in the anterior cingulate gyrus using ^1^H-magnetic resonance spectroscopy (^1^H-MRS), we observed a significant reduction in the NAA/Cr ratio (indicative of neuronal loss) at baseline. This corresponded to early features of cognitive impairment at a time when no changes in the NAA/Cr ratio were observed in the motor cortex in the region of hand representation (Strong et al., 1999). In hindsight, when we returned to these studies to correlate the placement of the STEAM (Stimulated Echo Acquisition Mode) voxel for mesial frontal localization, the localization was predominantly area 24, showing that the region of neuronal loss correlated with those regions described earlier as encoding social cognition and ToM. As will be reviewed below, it was the neuropathological study of these cases that has driven our conceptualization of a tauopathy in association with ALS-FTSD.

## ALS-FTSD: neuropathological characterization

### Alterations in RNA metabolism

Pathological intracellular protein inclusions are amongst the neuropathological hallmarks of ALS and include proteins such as copper/zinc superoxide dismutase 1 (SOD1) (Chou et al., 1996), TDP-43 (Arai et al., 2006; Neumann et al., 2006), fused in sarcoma/translocated in liposarcoma (FUS/TLS) (Kwiatkowski et al., 2009; Vance et al., 2009), TATA-binding protein-associated factor 15 (TAF-15) (Couthouis et al., 2011), Ewing sarcoma breakpoint region 1 (EWSR1) (Couthouis et al., 2011), RNA-binding motif 45 (RBM45) (Collins et al., 2012), Rho guanine nucleotide exchange factor (RGNEF) (Volkening et al., 2010; Keller et al., 2012), intermediate filament proteins (neurofilament Strong et al., 2005 or peripherin Migheli et al., 1993), 14-3-3 proteins (Malaspina et al., 2000; Kawamoto et al., 2004), and abnormal dipeptide repeat (DPR) proteins arising from *C9orf72 G*_4_*C*_2_ repeat expansions through repeat associated non-AUG (RAN) translation (Ash et al., 2013; Mori et al., 2013).

Amongst these proteins, it is the presence of TDP-43 immunoreactive neuronal cytoplasmic inclusions (NCIs) within degenerating motor neurons that is now recognized as being a ubiquitous neuropathological feature of all variants of ALS, with the exception of cases in which mutations in SOD1 are present. TDP-43 is a dual DNA/RNA binding protein whose metabolism is fundamentally altered in ALS and which forms the core protein of the vast majority of neuronal and glial inclusions in ALS, FTD, and ALS-FTSD (Arai et al., 2006; Neumann et al., 2006). Typically, TDP-43 is predominantly nuclear in its localization, but in response to cellular injury, its expression is markedly upregulated in a process that is accompanied by a nuclear to cytosolic shift in its localization (Moisse et al., 2009a,b). There is a high degree of correlation between the severity of the cognitive deficit that occurs in ALS and the distribution and extent of pathological TDP-43 deposition (Mackenzie, 2008; Mackenzie et al., 2011; Cykowski et al., 2017). Because of this prominence of TDP-43 pathology, and a general lack of deposition of the microtubule associated tau protein, the FTLD of ALS-FTD is currently classified as a “TDP-43 proteinopathy” and not a “tau proteinopathy.”

### pThr^175^ tau and alterations in tau metabolism

Notwithstanding this, the evidence in support of alterations in tau metabolism in ALS is robust, including that observed in the hyperendemic focus of ALS in the western Pacific in which the deposition of tau protein is prominent throughout the neuroaxis (Hirano, 1966). The presence of tau pathology has also been described in both sporadic and familial ALS case reports (Orrell et al., 1995; Soma et al., 2012; Dobson-Stone et al., 2013; Nakamura et al., 2014; Takeuchi et al., 2016). Additionally, several more recent studies have reported an elevation in phosphorylated and truncated tau protein in both hippocampal and spinal cord neurons of various ALS populations (Gómez-Pinedo et al., 2016; Vintilescu et al., 2016).

Our interest in the pathological processing of tau protein in ALS was initially aimed at explaining the basis of the superficial linear spongiosus evident in the anterior cingulate gyri of those ALSci patients in whom we observed a loss of neurons in this region through ^1^H-MRS (Strong et al., 1999; Wilson et al., 2001). Using antibodies against tau protein as a marker of axonal projections which we postulated would be lost, we instead observed both neuronal and glial cytoplasmic inclusions which were tau immunoreactive throughout the affected anterior cingulate gyrus (Yang et al., 2003). This included a broad range of pathologies, including neurofibrillary tangle-like structures, dystrophic neurites, neuritic granules, and tau-immunoreactive tufted astrocytes. We demonstrated that this was not simply a function of aging as might be expected based on the subsequently described progressive age-related tauopathy (Yang et al., 2005; Crary et al., 2014; Jellinger et al., 2015). Following fractionation of tau protein into sarkosyl-soluble or insoluble fractions from either the cortical or subcortical white matter of the anterior cingulate gyrus of ALSci patients, we observed that in contrast to tau protein isolated from Alzheimer's disease in which the insoluble fraction contained the triplet tau isoforms characteristic of paired helical filaments (PHFs), all six isoforms of tau protein were expressed in both the soluble and insoluble fractions (Strong et al., 2006). We subsequently confirmed this finding independent of the presence or absence of pathological hexanucleotide expansions of C9*orf* 72 (Volkening et al., 2017). In the former study, we also observed that sarkosyl-insoluble tau protein from ALSci was resistant to enzymatic dephosphorylation, and, when examined using the Thioflavin S assay, had a significant increase in the tendency to form polymers *ex vivo* (Strong et al., 2006).

**Figure 1:**
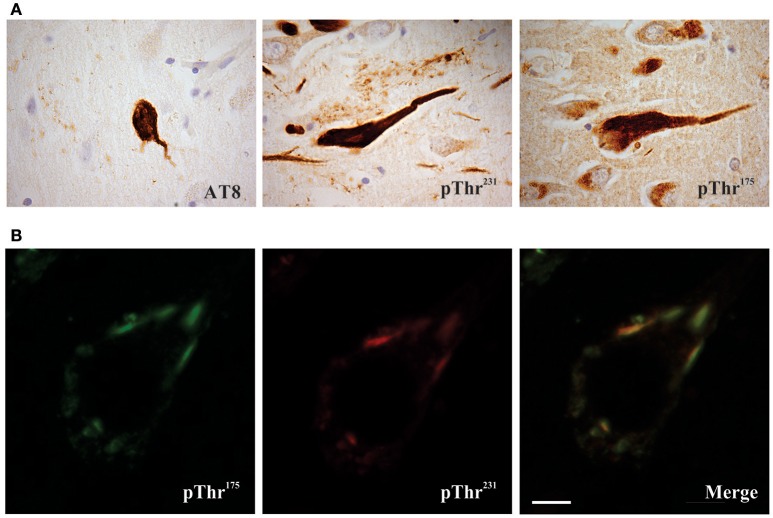
Phosphorylated tau pathology in amyotrophic latera sclerosis with cognitive impairment (ALSci) hippocampal neurons. **(A)** Immunohistochemical probing with AT8 (pSer^202^, pThr^205^), pThr^175^, pThr^231^ all reveal positive inclusions in ALS hippocampal neurons. Images taken with a 100x objective. **(B)** Representative image of pThr^175^ and pThr^231^ co-localization in ALSci hippocampal neuron. Scale bar = 5 μm.

Given this suggestion of pathological tau protein phosphorylation in ALSci, we conducted a phospho-epitope analysis and identified a unique tau phosphorylation site at Threonine 175 (pThr^175^ tau) in ALS and ALSci that was not present in either control or AD (both soluble and insoluble tau protein isolates) (Strong et al., 2006). Using a novel polyclonal antibody against pThr^175^ tau, we subsequently confirmed the presence of prominent tau protein glial and neuronal cytoplasmic inclusions in ALSci, and to a lesser degree in ALS without cognitive deficits, in association with an increase in the expression of active GSK3β (Yang et al., 2008; Yang and Strong, 2012) (Figure 1). This deposition was accompanied by a diffuse increase in TDP-43 immunoreactivity, suggesting the co-occurrence of these pathological processes (Yang and Strong, 2012). The prominent deposition of tau protein, including pThr^175^ tau, has been subsequently validated in motor neuron disease (MND) and MND in the presence of a behavioral variant of FTD (bvFTD-MND), including the appearance of tau protein deposition in 10% of MND cases that did not conform to traditional morphological criteria (unclassified frontal tau protein) (Behrouzi et al., 2016). While the latter study suggested a morphological appearance reminiscent of AD, as discussed earlier, the characteristic sarkosyl-insoluble tau protein triplet protein molecular signature of AD has not been observed in ALS or ALSci. More recently, we have observed that the pathological phospho-epitope pThr^175^ tau protein is also observed across a broad range of tauopathies, strongly suggesting that its presence in ALSci is not simply incidental, but rather a marker of a fundamental alteration in tau protein processing across a range of tauopathies (Moszczynski et al., 2017).

## Pathogenicity of pThr^175^ tau

To confirm the pathogenicity of pThr^175^ tau, we transfected either HEK293T or Neuro2a cells with a pseudo-phosphorylated tau protein construct (Thr^175^Asp-tau), wild type human tau protein (WT-tau) or human tau protein in which phosphorylation at Thr^175^ was irreversibly inhibited (Thr^175^Ala-tau) (Gohar et al., 2009). We observed a significant induction of pathological tau protein cytoplasmic inclusions and enhanced rates of cell death in the presence of Thr^175^Asp-tau, regardless of which of the six human tau protein constructs was transfected. Consistent with the observed up-regulation of activated GSK3β (pTyr^216^GSK3β) that co-localized with tau protein pathology in ALSci, we demonstrated a significant increase in pTyr^216^ GSK3β expression in only the Thr^175^Asp-tau transfected cells (Moszczynski et al., 2015). Inhibition of GSK3β activity, either pharmacologically or by shRNA against GSK3β, abolished inclusion formation. We next demonstrated that the development of pathological inclusions in response to the presence of Thr^175^Asp-tau was dependent on the unprimed phosphorylation of the Thr^231^ residue of tau protein and that the presence of pThr^231^ was sufficient to induce tau protein inclusions. These observations suggest that a pathway of pThr^175^ induction of GSK3β activation and a consequent phosphorylation of Thr^231^ in tau protein fibril formation is a pathogenic mechanism contributing to neuronal death in ALSci. While investigating evidence for this pathway in human disease, we observed the co-localization of pThr^175^ and pThr^231^ in tau protein NCI-containing hippocampal neurons in ALSci and a broad range of tauopathies including Alzheimer's disease, diffuse Lewy body dementia, and FTLD among others (Moszczynski et al., 2017). What remains to be determined across both sets of experiments however is the ultrastructural characteristics of the cytoplasmic inclusions driven *in vitro* by pThr^175^ tau or as observed *in vivo*.

We then examined the tauopathy associated with chronic traumatic encephalopathy (CTE) and CTE with concurrent ALS (CTE-ALS) (Moszczynski et al., 2018). In both disease states, the neuropathological hallmark is a disseminated tauopathy including not only cortical neuronal and glial cytoplasmic inclusions, but also spinal cord tau protein pathology (McKee et al., 2009, 2010; Mez et al., 2017). Consistent with a role for pathological Thr^175^ tau phosphorylation, we observed pThr^175^, pThr^231^, and T22 immunoreactivity in both hippocampal and spinal motor neurons. Fractionation of the hippocampal tau protein into sarkosyl-soluble and insoluble fractions yielded all six tau protein isoforms partitioning into both fractions, similar to that observed in ALSci. In support of the hypothesis that this pathway is directly related to the induction of tau protein cytoplasmic inclusions, we were able to replicate this pathology following a single cortical impact (moderate brain trauma model) in young adult Sprague Dawley rats (Moszczynski et al., 2018).

While we have suggested that pThr^175^ tau is critical to the induction of a tauopathy, this evidence does not prove it to be sufficient for the development of tauopathy *in vivo*. To test this, we utilized the somatic gene transfer of a pseudophosphorylated human tau protein construct mimicking pThr^175^ tau. Young adult Sprague Dawley rats received bilateral stereotactic inoculums of a rAAV9 adenoviral construct containing one of either GFP tagged Thr^175^Asp-tau (pThr^175^ tau mimic), GFP-tagged WT-tau, GFP-tagged construct alone, or GFP-tagged Thr^175^Ala-tau (Moszcyznski et al., 2017). At 1 year following inoculation, expression of the viral vector remained prominent as assessed using anti eGFP immunohistochemistry. However, the brains of the Thr^175^Asp-tau inoculated rats demonstrated prominent neuronal tau protein pathology, including corkscrew neurites, and tau immunoreactive neuronal cytoplasmic inclusions.

## Postulated pathogenic pathway

In support of the pathogenicity of pThr^175^ tau, phosphorylation at this site is not observed in fetal human tissue (which is normally hyperphosphorylated) (Kenessey and Yen, 1993) or tissue from healthy-aged individuals (Moszczynski et al., 2017). We have shown *in vitro* that a pseudophosphorylated human tau construct (Thr^175^Asp tau) induces pathological tau protein inclusions accompanied by the activation of GSK3β along with phosphorylation of tau at Thr^231^ (Moszczynski et al., 2015). The *in vivo* observation of co-expression of pThr^175^ tau, pTyr^216^GSK3β, pThr^231^ tau, and immunoreactivity to a tau protein antibody recognizing oligomeric tau (T22) strongly suggests that this pathway is critical to the induction of pathological tauopathies, including ALSci, CTE and CTE-ALS (Moszczynski et al., 2017, 2018).

The mechanism of Thr^175^ phosphorylation is not known. Candidate kinases include mitogen activated protein kinases (MAPK), GSK3β, p38, and leucine-rich repeat kinase 2 (LRRK2) (Hanger et al., 1998; Reynolds et al., 2000; Atzori et al., 2001) (see also https://docs.google.com/spreadsheets/d/1hGYs1ZcupmTnbB7n6qs1r_WVTXHt1O7NBLyKBN7EOUQ/edit#gid=0). Amongst these, a member of the MAPK family of kinases, c-Jun N-terminal kinase [JNK; also known as stress activated protein kinase (SAPK)], is of specific interest given its increased activity in response to various cellular stresses *in vitro* (Namgung and Xia, 2000) and in the acute phases following traumatic brain injury (TBI) *in vivo* (Tran et al., 2012). Additionally, the inhibition of JNK can reduce pathological tau protein phosphorylation following TBI in a rodent model (Tran et al., 2012).

**Figure 2:**
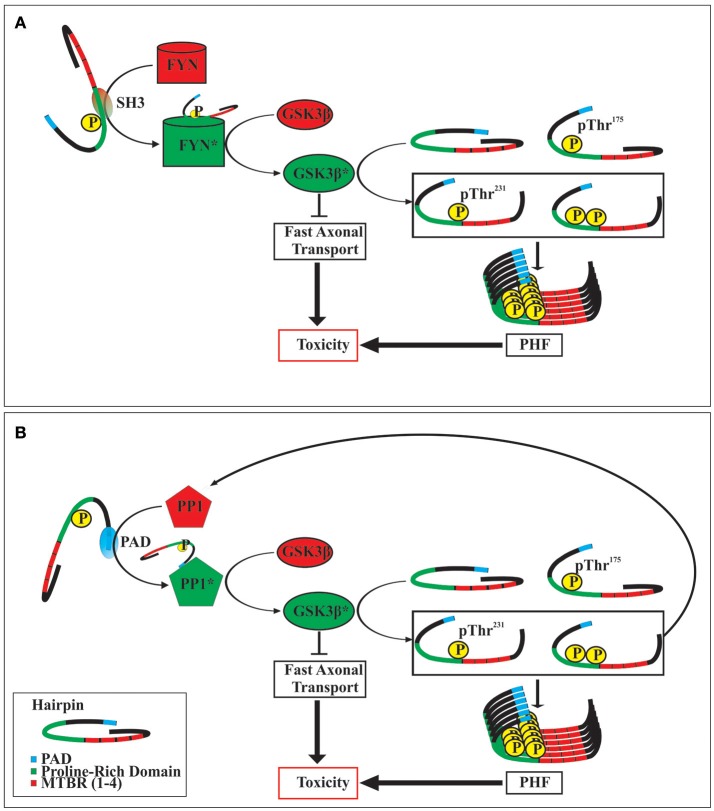
Two postulated mechanisms of toxicity following tau protein phosphorylation at Thr^175^. Opening of the tau hairpin conformation by phosphorylation at Thr^175^ exposes SH3 homology domains within the proline-rich region **(A)**. This interacts with and activates FYN kinase, which in turn activates GSK3β by phosphorylating at Tyr^216^. Alternatively, opening of the tau hairpin can expose the N-terminal phosphatase activating domain (PAD) **(B)**, which then activates protein phosphatase 1 (PP1). Activated PP1 dephosphorylates GSK3β at Ser^9^ increasing its activity. The activation of GSK3β may exert toxic effects in the cell by the inhibition of fast axonal transport, and by the phosphorylation of tau protein at Thr^231^, which promotes the formation of neurofibrillary tangles (paired helical filaments; PHF). Additionally, increased activity of GSK3β can increase the formation of pThr^231^ tau which also contains an exposed PAD, thereby initiating a dysregulated positive feedback loop. Activated enzymes are indicated by an asterisk (^*^).

Although the mechanism(s) by which the phosphorylation of tau at Thr^175^ leads to the activation of GSK3β are not yet known, it is likely that such mechanism(s) will involve altering tau's global hairpin structure given that Thr^175^ lies within the Pro-rich domain/hinge region of the hairpin as it has been shown that the phosphorylation of other residues within this domain can open the hairpin structure (Jeganathan et al., 2006, 2008). There are at least two potential mechanisms of GSK3β activation which may result from tau hairpin structure opening (Figure 2). Firstly, opening of the hairpin may expose the N-terminus proline-rich domain, which has been shown to interact with the SH3 domain of Src-family tyrosine kinase Fyn. In doing so, activated Fyn kinase may then phosphorylate GSK3β at the Tyr^216^ residue thereby enhancing its activity (Lee et al., 1998; Lesort et al., 1999; Klein et al., 2002). Secondly, opening of the hairpin conformation has been shown to expose the N-terminal phosphatase activating domain (PAD), consisting of amino acids 2–18. Exposure of this domain leads to activation of protein phosphatase-1 (PP1) which then dephosphorylates GSK3β at the Ser^9^ residue, enhancing GSK3β activity (Kanaan et al., 2011). In keeping with this mechanism, exposure of the PAD has been suggested to be important in tau protein mediated neuronal toxicity through interference with fast axonal transport, and may be an early event in the development of a number of tauopathies (Kanaan et al., 2011, 2016; Ward et al., 2012; Combs et al., 2016).

In the studies cited above, tau phosphorylation at Thr^231^ is a critical step in the induction of pathological fibrils. Several mechanisms have been proposed by which this may occur, with alterations in tau degradation being key in a process that is dependent on tau conformation. Of note, *cis*-pThr^231^ tau demonstrates specificity in the development of pathology over *trans*-pThr^231^ tau, in which the *cis* conformer is pathological and cannot be degraded, whereas the *trans* conformer is physiological and can be degraded (Nakamura et al., 2012). The isomerase, peptidyl-prolyl *cis*-*trans* isomerase NIMA-interacting 1 (PIN1), converts the pathologic *cis* conformer to the physiologic *trans* conformer, allowing for dephosphorylation at this site by protein phosphatase 1 (Lu et al., 1996, 1999). It is of note, therefore, that *cis*-pThr^231^ tau is closely associated with a number of tauopathies including, AD, CTE and experimental CTE (Lu et al., 1996, 1999; Liou et al., 2003; Pastorino et al., 2006; Sultana et al., 2006; Lu and Zhou, 2007; Lim et al., 2008; Lee et al., 2011; Chen et al., 2015; Kondo et al., 2015; Albayram et al., 2017). Given that both *cis*-pThr^231^ tau and pThr^175^ tau are observed exclusively in disease or stressed states, it is attractive to postulate that the phosphorylation of tau protein at Thr^175^ increases the presence of *cis*-pThr^231^. This may occur through either enhancing the probability of *cis* phosphorylation by GSK3β or by inhibiting the ability of PIN1 to isomerize the epitope from *cis* to *trans*. This is the subject of current studies.

## The potential role of comorbid pathologies

The fact that other cell stressors may induce Thr^175^ tau protein phosphorylation is of great significance to the mechanisms of neurodegenerative disease. Pathologies are rarely, if ever, observed in isolation, and it is common to identify co-morbid pathologies in the same brain (Amador-Ortiz et al., 2007; Josephs et al., 2014a,b; Smith, 2017). While this high incidence of comorbidity has traditionally been attributed to the aging process, it is increasingly evident that multiple pathologies are present even in young cohorts of patients afflicted with neurodegenerative disease. Therefore, there is likely an interplay between pathological processes that drives these co-morbid pathologies (Spires-Jones et al., 2017; Tan et al., 2017). As such, the possibility of synergistic toxicity must be considered when trying to understand the mechanisms that drive neurodegenerative disease, and when attempting to treat such diseases.

As discussed earlier, the FTLD of ALSci is classified as a FTLD-TDP based on the prominence of pathological TDP-43 deposition. The association of TDP-43 pathology with ALS and ALS-FTSD is well described (Arai et al., 2006; Neumann et al., 2006). However, there is also an increasing literature describing comorbid TDP-43 and tau pathologies, such as that observed in AD and CTE (McKee et al., 2010; Josephs et al., 2014a,b). The relationship between tau protein and TDP-43 pathology is strengthened in the observation that the effective elimination of aberrantly phosphorylated tau protein in a rodent model of CTE can also lead to the prevention of TDP-43 pathology (Albayram et al., 2017). It is possible that one pathological process primes the other. In this case, the toxicity of pseudophosphorylated Thr^175^Asp tau protein expression may be enhanced by the co-expression of TDP-43, serving as a second hit to the CNS. The potential of this synergistic toxicity is the focus of ongoing studies in our lab.

## Summary

The presence of pathological tau processing is clearly evident across a broad range of ALS patients—including those found within the hyperendemic foci such as that seen in the western Pacific, a smattering of case reports, and ALSci as well as FTD-MND. This suggests that this is not a simple matter of incidental co-occurrence. Moreover, there is solid evidence to support the pathogenic role of pThr^175^ in the induction of a tauopathy, both *in vitro* and *in vivo*, and the pathological cascade induced by pThr^175^ which culminates in the generation of oligomeric tau has been observed in a broad range of tauopathies. The issue is therefore not whether a tauopathy exists in ALS-FTSD, but rather the extent to which it is the driving pathology—either alone, or in combination with the hallmark pathology of TDP-43—of the clinical and neuropathological phenotype.

Such a proposal however leaves many unanswered questions, including: the mechanism by which the Thr^175^ residue is initially phosphorylated; how pThr^175^ tau leads to the activation of GSK3β; whether pThr^231^ tau is necessary and sufficient to induce neuronal dysfunction and death, or whether additional tau protein pathological phosphorylation is needed; and what (if any) role for the co-existent TDP-43 pathology of ALS-FTSD is also necessary to drive the phenomenological aspects of pThr^175^ tau-mediated tauopathy.

## Author contributions

MS conceived and wrote the article. AM conceived and wrote the article. MH wrote the article.

### Conflict of interest statement

The authors declare that the research was conducted in the absence of any commercial or financial relationships that could be construed as a potential conflict of interest.
